# Transcutaneous Spinal Direct Current Stimulation

**DOI:** 10.3389/fpsyt.2012.00063

**Published:** 2012-07-04

**Authors:** Filippo Cogiamanian, Gianluca Ardolino, Maurizio Vergari, Roberta Ferrucci, Matteo Ciocca, Emma Scelzo, Sergio Barbieri, Alberto Priori

**Affiliations:** ^1^Unità Operativa di Neurofisiopatologia, Fondazione IRCCS Ca’ Granda Ospedale Maggiore PoliclinicoMilan, Italy; ^2^Centro Clinico per la Neurostimolazione, le Neurotecnologie ed i Disordini del Movimento, Fondazione IRCCS Ca’ Granda Ospedale Maggiore PoliclinicoMilan, Italy; ^3^Dipartimento di Scienze Neurologiche, Università degli Studi di MilanoMilan, Italy; ^4^Unità Operativa di Neurologia, Fondazione IRCCS Ca’ Granda Ospedale Maggiore PoliclinicoMilan, Italy

**Keywords:** transcranial direct current stimulation, transcutaneous spinal direct current stimulation, spinal cord, pain

## Abstract

In the past 10 years renewed interest has centered on non-invasive transcutaneous weak direct currents applied over the scalp to modulate cortical excitability (“brain polarization” or transcranial direct current stimulation, tDCS). Extensive literature shows that tDCS induces marked changes in cortical excitability that outlast stimulation. Aiming at developing a new, non-invasive, approach to spinal cord neuromodulation we assessed the after-effects of thoracic transcutaneous spinal DC stimulation (tsDCS) on somatosensory potentials (SEPs) evoked in healthy subjects by posterior tibial nerve (PTN) stimulation. Our findings showed that thoracic anodal tsDCS depresses the cervico-medullary PTN-SEP component (P30) without eliciting adverse effects. tsDCS also modulates post-activation H-reflex dynamics. Later works further confirmed that transcutaneous electric fields modulate spinal cord function. Subsequent studies in our laboratory showed that tsDCS modulates the flexion reflex in the human lower limb. Besides influencing the laser evoked potentials (LEPs), tsDCS increases pain tolerance in healthy subjects. Hence, though the underlying mechanisms remain speculative, tsDCS modulates activity in lemniscal, spinothalamic, and segmental motor systems. Here we review currently available experimental evidence that non-invasive spinal cord stimulation (SCS) influences spinal function in humans and argue that, by focally modulating spinal excitability, tsDCS could provide a novel therapeutic tool complementary to drugs and invasive SCS in managing various pathologic conditions, including pain.

## Introduction

In the past two decades growing interest has centered on non-invasive brain stimulation techniques such as transcranial magnetic stimulation (TMS) and transcranial direct current stimulation (tDCS). Extensive literature shows that tDCS can modulate activity in specific cerebral cortex regions by inducing marked changes in cortical excitability that outlast stimulation (Nitsche et al., 2003a; Priori, 2003; Paulus, 2004; Priori et al., 2009; Stagg and Nitsche, 2011). Thanks to its low costs, acceptable safety data and potential use in outpatients, tDCS is increasingly being evaluated in proof-of-principle and pivotal clinical trials in widely ranging neurological and psychiatric disorders (Murphy et al., 2009; Nitsche et al., 2009; Baker et al., 2010; O’connell et al., 2011; Schlaug et al., 2011). Numerous studies have addressed the physiological effects induced by tDCS on the cerebral cortex and evidence comes from stimulation applied to the primary motor cortex (M1; to review Stagg and Nitsche, 2011). tDCS can modulate cortical excitability and neuronal firing rates. Direct current stimulation changes the resting neuronal membrane potential in the cortex layers (Bindman et al., 1964; Purpura and McMurtry, 1965). Depending on the duration and strength of polarization, these changes can persist after stimulation offset. Long-lasting effects after brain polarization probably arise through synaptic changes via long-term potentiation (LTP) and depression (LTD; Liebetanz et al., 2002; Nitsche et al., 2003a) as well as non-synaptic mechanisms (Ardolino et al., 2005).

Surprisingly, rediscovering the use of direct currents on the brain has not yet prompted a similar effort to explore the possibility of using non-invasive, transcutaneous, direct current stimulation to modulate spinal cord function. Having a technique for modulating spinal function is important for various reasons. First, several neurologic diseases and syndromes arise from an acquired or congenital selective spinal cord dysfunction. Given that the brain and spinal cord interact through several projections and that DC stimulation over the spine may modulate different supraspinal activities, transcutaneous spinal DC stimulation (tsDCS) has numerous clinical applications. Finally, invasive electrical spinal cord stimulation (SCS) has been used for more than 30 years to treat a variety of pain syndromes. Traditionally used for persisting leg pain after lumbar spinal surgery, SCS has been applied successfully in the treatment of angina pectoris, ischemic pain in the extremity and complex regional pain syndromes (Grabow et al., 2003; Mailis-Gagnon et al., 2004; Ubbink and Vermeulen, 2005; Frey et al., 2009). The aim of this paper is to review studies on the use of tsDCS in humans focusing on the technique’s physiological effects and potential clinical applications. The first step in conducting the review involved a selective literature search for papers published from 1990 to March 2012. We used the PUBMED of the National Library of Medicine database. Our key search terms were “direct current stimulation” or “tsDCS” or “polarization” and “spinal cord” with the limitation that studies were written in English.

## Effects of tsDCS on Spinal Tracts

### Lemniscal tract

To investigate whether transcutaneous direct currents can interfere with ascending somatosensory pathways in the human spinal cord, in a study from our group we evaluated the after-effects induced by anodal and cathodal tsDCS on somatosensory potentials (SEPs) evoked by stimulating the posterior tibial nerves (PTN; Cogiamanian et al., 2008). We applied current at a density of 0.071 mA/cm^2^ and delivered a total charge of 63.9 mC/cm^2^ (2.5 mA for 15 min, electrode area 35 cm^2^) with the active electrode located on the thoracic spinal cord (over the spinous process of the tenth thoracic vertebra) and the reference above the right shoulder. Anodal tsDCS selectively reduced the amplitude of the cervico-medullary component (P30) of PTN-SEPs for at least 20 min after stimulation offset. Conversely cathodal tsDCS left P30 almost unchanged in amplitude.

By analogy with the effects of DC currents on peripheral nerve axons, we hypothesized that anodal currents hyperpolarize sensory axons running in the posterior columns of the spinal cord ultimately leading to an “anodal block” (Bhadra and Kilgore, 2004). Interestingly whereas anodal tsDCS decreased amplitudes it left latencies unchanged. This finding agrees with the observation that also at peripheral nerve level anodal polarization decreases the size of the motor responses, but not the latency (Priori et al., 2005). Anodal tsDCS could fail to induce latency changes because it blocks impulse conduction in some axons, leaving conduction in the remaining axons unaltered. Our data were indirectly confirmed in a subsequent study by Aguilar et al. (2011) in anesthetized rats. These authors investigated how direct current spinal stimulation delivered at thoracic level influences spontaneous activity and SEPs in the gracile nucleus and primary somatosensory cortex. They used a different stimulation setup with one electrode placed on the thoracic spinal cord over the exposed dura mater, and the second under the skin in the anterior abdominal area aiming to maximize the current focus in the spinal cord below the dorsal electrode. Anodal spinal direct current stimulation (sDCS) increased spontaneous activity in the gracile nucleus while decreasing its local field potentials responses to somatosensory stimuli, and cathodal sDCS did the opposite. This inverse relationship between gracile spontaneous activity and local field potentials, the equivalent of SEPs at brainstem level (P30), depended on several mechanisms including pre-synaptic inhibition, synaptic depression, and shunting inhibition (Aguilar et al., 2011).

### Spinothalamic tract

Given this basic ability of tsDCS to modulate conduction in the lemniscal pathway, Truini et al. (2011) sought further information by evaluating the effects of thoracic tsDCS (2.5 mA, 20 min) on the spino-thalamic tract. To do so, they investigated the after-effects of anodal and cathodal tsDCS delivered on the skin overlying the thoracic spinal cord on foot and perioral laser evoked potentials (LEPs) in a group of healthy subjects. Peripheral laser stimulation selectively activates Aδ and C mechano-thermal nociceptors (Treede et al., 1995), and evokes scalp potentials related to small myelinated (Aδ) fibers (Romaniello et al., 2003). Using an electrode set-up (active electrode on the thoracic spinal cord on the skin over the thoracic spinous process of the tenth thoracic vertebra and the reference above the right shoulder) as well as a stimulation protocol (2.5 mA, 20 min) similar to those we used in our earlier study Cogiamanian et al. (2008) and Truini et al. (2010) showed that anodal tsDCS reduced LEPs amplitude after foot stimulation whereas cathodal polarity induced a slight non-significant attenuation over time. Neither anodal nor cathodal tsDCS changed LEP variables after perioral stimulation suggesting that the DC-induced changes took place at spinal level. In an additional experiment to better understand the behavioral significance of these findings on LEPs the same investigators tested the effects of thoracic tsDCS on the foot-cold-pressor test, a pain model that has been widely used in human pain research. The foot-cold-pressor test analysis disclosed higher pain tolerance during anodal than during cathodal tsDCS. Conversely, no significant difference was found in the pain threshold between the two polarity conditions. The lack of tsDCS effects on LEPs and cold-pressor test thresholds was related to a poor sensitivity of these variables as minimal afferent input could be sufficient to maintain them normal (Truini et al., 2010).

## Effects of tsDCS on Spinal Reflexes

Besides its ability to modulate the progression of sensory or nociceptive inputs along the spinal cord tsDCS could also modulate spinal cord activity at segmental level. Two studies evaluated how transcutaneously applied direct currents influence spinal circuitries. In the first, Winkler et al. (2010) focused on tsDCS-induced changes in H-reflex size and post-activation H-reflex depression (or homosynaptic depression) namely, reduced H-reflex amplitude within 8–12 s after Ia fibers afferent activation. Earlier evidence already showed that reduced synaptic efficacy is related to decreased neurotransmitter release and exclusively affects the previously activated Ia fiber-motoneuron synapse with no effects due to supraspinal influences (Grey et al., 2008). tsDCS was applied using a pair of self-adhesive electrodes (40 cm^2^), the active one placed about 2 cm left paravertebrally to the 11th thoracic vertebra and the other in the left infraclavicular region. Direct current was administered for 15 min at an intensity of 2.5 mA, resulting in a current density of 0.063 mA/cm^2^ and a total delivered charge of 0.056 C/cm^2^.

General H-reflex excitability, measured with the Hmax/Mmax ratio, remained statistically unchanged after stimulation but anodal tsDCS induced a long-lasting decrease in H-reflex post-activation depression and cathodal tsDCS increased it. The investigators suggest that stimulation modulated efficiency in the Ia fiber-motoneuron synapse without influencing excitability in the alpha-motoneuron (Winkler et al., 2010).

In our laboratory we evaluated changes induced by thoracic tsDCS on the lower limb flexion reflex (LL-Fr) in 11 healthy subjects (Cogiamanian et al., 2011). The Fr is a polysynaptic spinal reflex elicited by electrical stimulation applied to a sensory nerve and is a reliable and widely investigated neurophysiologic tool to assess the efficacy of analgesic therapies (Cruccu et al., 2004). The Fr comprises an early response, the RII reflex (RIIr) and a late response, the RIII reflex (RIIIr). Various studies have shown that the RIIr is a non-nociceptive Aβ fiber mediated response, whereas the RIIIr is a high-threshold nociceptive Aδ fiber mediated reflex. The RIIIr threshold corresponds to the pain threshold and the size of the reflex is related to the pain perception level (Sandrini et al., 2005). Subjects underwent anodal tsDCS (2 mA, 15 min) and sham stimulation in a cross-over design with active (anodal) electrodes placed over the spinal process of the tenth thoracic vertebra and the cathode (reference) above the right shoulder. Thoracic anodal tsDCS induced a long-lasting Fr depression and reduced the RIII area by 27%. Both changes lasted for at least 30 min after stimulation offset. Because stimulation left H reflex variables unchanged we exclude the possibility that tsDCS inhibits the nociceptive reflex by modulating excitability in the mono-oligosynaptic segmental reflex pathway. These data along with the changes induced by tsDCS on foot-LEPs (Truini et al., 2011) reflect an attenuation of spinal processing of nociceptive inputs. Even though the exact mechanism through which tsDCS acts remains elusive it seems unrelated to the “gate theory of pain” advocated by Melzack and Wall (1965) to explain the analgesic effects of invasive epidural SCS. This theory proposed that the position of the “gate” depends upon the degree of large (non-painful) or small (painful) nerve fiber firing. When stimulation activates faster large fibers (as does SCS) the gate closes so that no impulses can pass through, thus eliminating or reducing pain. Conversely, when it predominantly activates small nerve fibers, pain messages can be transmitted. Placing the active anodal electrode on the thoracic spine in tsDCS experiments makes it unlikely that tsDCS directly activates Aβ fibers (closing of the gate) ascending from the foot. Equally important, a fundamental concept of SCS is that the analgesic effects rely on a sustained and strictly homotopic input that the subject must perceive on the projection territory (Oakley and Prager, 2002). Ample evidence shows that tsDCS induces occasional, transient, and short-lasting tingling and burning sensations just below the stimulating electrodes and that DCS strength remains below the conscious sensory threshold throughout the experimental sessions (Cogiamanian et al., 2008, 2011; Truini et al., 2011).

Similarly, we can exclude the possibility that tsDSC induces its modulatory effects by a specifically activating diffuse noxious inhibitory controls (DNIC). In our experiments (Cogiamanian et al., 2011) after anodal skin stimulation away from the spinal cord producing the same itching sensation as the original experiment, LL-Fr variables remained unchanged. In addition, Truini et al. (2011) showed that thoracic tsDCS had no effect on LEPs evoked by perioral stimulation (exceeding the tsDCS stimulation level).

From current knowledge we therefore hypothesize that tsDCS acts at spinal level. In humans nociception is mediated by a complex interneuronal network that integrates peripheral inputs, multisensory feedback, and supraspinal descending projections. Animal models of experimental mononeuropathy show that, in response to sciatic nerve lesions, multireceptive, wide dynamic range (WDR) neurons in the deeper lamina of the rat dorsal horn increase their spontaneous firing rates and exhibit after-discharge behavior that is attenuated by SCS (Dubuisson, 1989). tsDCS could interfere with the ascending nociceptive spinal pathway by reducing the “gain” in spinal nociceptive information transmission by modulating activity in the spinal interneuronal network. tsDCS could mediate this effect by directly activating segmental interneurons (i.e., WDR neurons) or by modulating dorsal column transmission via collaterals to dorsal horns. Alternatively, tsDCS could activate supraspinal loops, relayed by the brainstem or thalamocortical systems, thereby providing both ascending and descending inhibition.

## Safety Considerations

Even though tDCS and tsDCS are non-invasive techniques for neuromodulation and are commonly considered safe, caution is required. The use of tDCS in therapeutic protocols to date has not resulted in severe adverse effects, but some safety issues remain controversial. In an early study, Nitsche et al. (2003b) suggested that the appropriate variables for determining safety limits for tDCS should be current density (CD, mA/cm^2^) and total charge (TC, C/cm^2^). Data from the literature suggests that tissue damage occurs at a TC of 216 C/cm^2^ (Yuen et al., 1981) and that a CD below 25 mA/cm^2^ (McCreery et al., 1990) induces no tissue damage. Notably, the stimulation variables commonly used are a thousand-fold lower than these limits. When it begins and after it ends, tDCS often elicits short-lasting tingling sensations, rarely accompanied by redness under the electrode sites.

Safety data for tsDCS are scanty. No spinal-specific adverse events have been reported after tsDCS and we excluded direct harmful effects of tsDCS over spinal cord by assaying serum neuron specific enolase (NSE) before and immediately after stimulation offset (Cogiamanian et al., 2008). Although the stimulation variables that have been used in tsDCS protocols were comparable with those used in tDCS studies, we cannot exclude harmful effects due to a high local current density related, for instance, to current flow via the spinal foramina.

For future studies, patients undergoing tsDCS should be carefully monitored for adverse effects with conventional magnetic resonance imaging (MRI) or spectroscopy, because safety issues related to tsDCS may emerge only with larger studies or using novel stimulation protocols with repetitive daily sessions.

## Conclusion

The few papers published over the past 5 years we review here provide ample evidence that tsDCS induces changes in spinal cord function. The physiological mechanisms underlying these changes need further investigation. Because, unlike the brain, no methodology is available for non-invasive spinal neuromodulation, the possibility of influencing conduction along the ascending spinal pathways in humans is interesting, especially for clinical purposes.

Although its basic ability to modulate several neurophysiologic variables does not guarantee that tsDCS is effective as a clinical technique, because it induces no adverse effects, is simple and non-invasive, the findings from this review open the way to new approaches using non-invasive tsDCS for treating disorders that are presently managed with invasive methods. The widespread use of high-frequency epidural electrical stimulation to treat various chronic pain syndromes has prompted research to investigate whether tsDCS could be used to modulate nociception with a new non-invasive approach. tsDCS also promises to be useful in neurorehabilitation, especially in treating spasticity.

A major drawback that limits tsDCS for clinical use is that DC applied to single brain areas or to the spine induce after-effects that persist only for several minutes to several hours. Some help in prolonging the beneficial effects induced by tsDCS could come from optimizing stimulation protocols and devices. Various therapeutic protocols can be used to prolong the neuromodulatory effect of tsDCS. For example, patients can undergo repetitive sessions (i.e., daily sessions for more than two consecutive days) increasing the total charge administered. Portable tsDCS devices that outpatients can be trained to use daily or in repeated sessions are already available.

## Conflict of Interest Statement

Filippo Cogiamanian, Roberta Ferrucci, Maurizio Vergari, and Alberto Priori are stakeholders of Newronika s.r.l., a spin-off company of the Fondazione IRCCS Ca’ Granda Ospedale Maggiore Policlinico and of the Università degli Studi di Milano. Gianluca Ardolino, Matteo Ciocca, and Emma Scelzo have reported no conflicts of interest.

## References

[B1] Aguilar J. Pulecchi F. Dilena R. Oliviero A. Priori A. Foffani G. (2011). Spinal direct current stimulation modulates the activity of gracile nucleus and primary somatosensory cortex in anaesthetized rats. J. Physiol. (Lond.) 589, 4981–4996 21825031 10.1113/jphysiol.2011.214189 PMC3224887

[B2] Ardolino G. Bossi B. Barbieri S. Priori A. (2005). Non-synaptic mechanisms underlie the after-effects of cathodal transcutaneous direct current stimulation of the human brain. J. Physiol. (Lond.) 568, 653–663 10.1113/jphysiol.2005.088310 16037080 PMC1474743

[B3] Baker J. M. Rorden C. Fridriksson J. (2010). Using transcranial direct-current stimulation to treat stroke patients with aphasia. Stroke 41, 1229–1236 10.1161/STROKEAHA.109.569764 20395612 PMC2876210

[B4] Bhadra N. Kilgore K. L. (2004). Direct current electrical conduction block of peripheral nerve. IEEE Trans. Neural Syst. Rehabil. Eng. 12, 313–324 10.1109/TNSRE.2004.834205 15473193

[B5] Bindman L. J. Lippold O. C. Redfearn J. W. (1964). The action of brief polarizing currents on the cerebral cortex of the rat (1) during current flow and (2) in the production of long-lasting after-effects. J. Physiol. (Lond.) 172, 369–382 14199369 10.1113/jphysiol.1964.sp007425 PMC1368854

[B6] Cogiamanian F. Vergari M. Pulecchi F. Marceglia S. Priori A. (2008). Effect of spinal transcutaneous direct current stimulation on somatosensory evoked potentials in humans. Clin. Neurophysiol. 119, 2636–2640 10.1016/j.clinph.2008.07.249 18786856

[B7] Cogiamanian F. Vergari M. Schiaffi E. Marceglia S. Ardolino G. Barbieri S. Priori A. (2011). Transcutaneous spinal cord direct current stimulation inhibits the lower limb nociceptive flexion reflex in human beings. Pain 152, 370–375 10.1016/j.pain.2010.10.041 21159430

[B8] Cruccu G. Anand P. Attal N. Garcia-Larrea L. Haanpaa M. Jorum E. Serra J. Jensen T. S. (2004). EFNS guidelines on neuropathic pain assessment. Eur. J. Neurol. 11, 153–162 10.1111/j.1468-1331.2004.00791.x 15009162

[B9] Dubuisson D. (1989). Effect of dorsal-column stimulation on gelatinosa and marginal neurons of cat spinal cord. J. Neurosurg. 70, 257–265 10.3171/jns.1989.70.2.0257 2913223

[B10] Frey M. E. Manchikanti L. Benyamin R. M. Schultz D. M. Smith H. S. Cohen S. P. (2009). Spinal cord stimulation for patients with failed back surgery syndrome: a systematic review. Pain Physician 12, 379–397 19305486

[B11] Grabow T. S. Tella P. K. Raja S. N. (2003). Spinal cord stimulation for complex regional pain syndrome: an evidence-based medicine review of the literature. Clin. J. Pain 19, 371–383 10.1097/00002508-200311000-00005 14600537

[B12] Grey M. J. Klinge K. Crone C. Lorentzen J. Biering-Sorensen F. Ravnborg M. Nielsen J. B. (2008). Post-activation depression of soleus stretch reflexes in healthy and spastic humans. Exp. Brain Res. 185, 189–197 10.1007/s00221-007-1142-6 17932663

[B13] Liebetanz D. Nitsche M. A. Tergau F. Paulus W. (2002). Pharmacological approach to the mechanisms of transcranial DC-stimulation-induced after-effects of human motor cortex excitability. Brain 125, 2238–2247 10.1093/brain/awf238 12244081

[B14] Mailis-Gagnon A. Furlan A. D. Sandoval J. A. Taylor R. (2004). Spinal cord stimulation for chronic pain. Cochrane Database Syst. Rev. 3, CD003783 15266501 10.1002/14651858.CD003783.pub2

[B15] McCreery D. B. Agnew W. F. Yuen T. G. Bullara L. (1990). Charge density and charge per phase as cofactors in neural injury induced by electrical stimulation. IEEE Trans. Biomed. Eng. 37, 996–1001 10.1109/10.102812 2249872

[B16] Melzack R. Wall P. D. (1965). Pain mechanisms: a new theory. Science 150, 971–979 10.1126/science.150.3699.971 5320816

[B17] Murphy D. N. Boggio P. Fregni F. (2009). Transcranial direct current stimulation as a therapeutic tool for the treatment of major depression: insights from past and recent clinical studies. Curr. Opin. Psychiatry 22, 306–311 10.1097/YCO.0b013e32832a133f 19339889

[B18] Nitsche M. A. Boggio P. S. Fregni F. Pascual-Leone A. (2009). Treatment of depression with transcranial direct current stimulation (tDCS): a review. Exp. Neurol. 219, 14–19 10.1016/j.expneurol.2009.03.038 19348793

[B19] Nitsche M. A. Fricke K. Henschke U. Schlitterlau A. Liebetanz D. Lang N. Henning S. Tergau F. Paulus W. (2003a). Pharmacological modulation of cortical excitability shifts induced by transcranial direct current stimulation in humans. J. Physiol. (Lond.) 553, 293–301 10.1113/jphysiol.2003.049916 12949224 PMC2343495

[B20] Nitsche M. A. Liebetanz D. Lang N. Antal A. Tergau F. Paulus W. (2003b). Safety criteria for transcranial direct current stimulation (tDCS) in humans. Clin. Neurophysiol. 114, 2220–2222; author reply 2222–2223. 10.1016/S1388-2457(03)00235-9 14580622

[B21] Oakley J. C. Prager J. P. (2002). Spinal cord stimulation: mechanisms of action. Spine 27, 2574–2583 10.1097/00007632-200211150-00034 12435996

[B22] O’connell N. E. Wand B. M. Marston L. Spencer S. Desouza L. H. (2011). Non-invasive brain stimulation techniques for chronic pain. A report of a Cochrane systematic review and meta-analysis. Eur. J. Phys. Rehabil. Med. 47, 309–326 21494222

[B23] Paulus W. (2004). Outlasting excitability shifts induced by direct current stimulation of the human brain. Suppl. Clin. Neurophysiol. 57, 708–714 10.1016/S1567-424X(09)70411-8 16106673

[B24] Priori A. (2003). Brain polarization in humans: a reappraisal of an old tool for prolonged non-invasive modulation of brain excitability. Clin. Neurophysiol. 114, 589–595 10.1016/S1388-2457(03)00236-0 12686266

[B25] Priori A. Bossi B. Ardolino G. Bertolasi L. Carpo M. Nobile-Orazio E. Barbieri S. (2005). Pathophysiological heterogeneity of conduction blocks in multifocal motor neuropathy. Brain 128, 1642–1648 10.1093/brain/awh513 15888541

[B26] Priori A. Hallett M. Rothwell J. C. (2009). Repetitive transcranial magnetic stimulation or transcranial direct current stimulation? Brain Stimul. 2, 241–245 10.1016/j.brs.2009.02.004 20633424

[B27] Purpura D. P. McMurtry J. G. (1965). Intracellular activities and evoked potential changes during polarization of motor cortex. J. Neurophysiol. 28, 166–185 14244793 10.1152/jn.1965.28.1.166

[B28] Romaniello A. Iannetti G. D. Truini A. Cruccu G. (2003). Trigeminal responses to laser stimuli. Neurophysiol. Clin. 33, 315–324 10.1016/j.neucli.2003.10.010 14678845

[B29] Sandrini G. Serrao M. Rossi P. Romaniello A. Cruccu G. Willer J. C. (2005). The lower limb flexion reflex in humans. Prog. Neurobiol. 77, 353–395 10.1016/j.pneurobio.2005.11.003 16386347

[B30] Schlaug G. Marchina S. Wan C. Y. (2011). The use of non-invasive brain stimulation techniques to facilitate recovery from post-stroke aphasia. Neuropsychol. Rev. 21, 288–301 10.1007/s11065-011-9181-y 21842404 PMC3176334

[B31] Stagg C. J. Nitsche M. A. (2011). Physiological basis of transcranial direct current stimulation. Neuroscientist 17, 37–53 10.1177/1073858410386614 21343407

[B32] Treede R. D. Meyer R. A. Raja S. N. Campbell J. N. (1995). Evidence for two different heat transduction mechanisms in nociceptive primary afferents innervating monkey skin. J. Physiol. (Lond.) 483(Pt 3), 747–758 7776255 10.1113/jphysiol.1995.sp020619 PMC1157815

[B33] Truini A. Panuccio G. Galeotti F. Maluccio M. R. Sartucci F. Avoli M. Cruccu G. (2010). Laser-evoked potentials as a tool for assessing the efficacy of antinociceptive drugs. Eur. J. Pain 14, 222–225 10.1016/j.ejpain.2009.05.001 19477145 PMC4878893

[B34] Truini A. Vergari M. Biasiotta A. La Cesa S. Gabriele M. Di Stefano G. Cambieri C. Cruccu G. Inghilleri M. Priori A. (2011). Transcutaneous spinal direct current stimulation inhibits nociceptive spinal pathway conduction and increases pain tolerance in humans. Eur. J. Pain 15, 1023–1027 10.1016/j.ejpain.2011.04.009 21576030

[B35] Ubbink D. T. Vermeulen H. (2005). Spinal cord stimulation for non reconstructable chronic critical leg ischaemia. Cochrane Database Syst. Rev. 3, CD004001 12917998 10.1002/14651858.CD004001

[B36] Winkler T. Hering P. Straube A. (2010). Spinal DC stimulation in humans modulates post-activation depression of the H-reflex depending on current polarity. Clin. Neurophysiol. 121, 957–961 10.1016/j.clinph.2010.01.014 20153248

[B37] Yuen T. G. Agnew W. F. Bullara L. A. Jacques S. McCreery D. B. (1981). Histological evaluation of neural damage from electrical stimulation: considerations for the selection of parameters for clinical application. Neurosurgery 9, 292–299 10.1227/00006123-198109000-00013 7301072

